# Molecular Cloning and Characterization of a Novel *α*-Amylase from Antarctic Sea Ice Bacterium* Pseudoalteromonas *sp. M175 and Its Primary Application in Detergent

**DOI:** 10.1155/2018/3258383

**Published:** 2018-06-27

**Authors:** Xiaofei Wang, Guangfeng Kan, Xiulian Ren, Geng Yu, Cuijuan Shi, Qiuju Xie, Hua Wen, Michael Betenbaugh

**Affiliations:** ^1^School of Marine Science and Technology, Harbin Institute of Technology at Weihai, Weihai 264209, China; ^2^School of Chemistry and Chemical Engineering, Harbin Institute of Technology, Harbin 150001, China; ^3^Department of Chemical and Biomolecular Engineering, Johns Hopkins University, Baltimore 21218, USA

## Abstract

A novel cold-adapted and salt-tolerant *α*-amylase gene (*amy*175) from Antarctic sea ice bacterium* Pseudoalteromonas *sp. M175 was successfully cloned and expressed. The open reading frame (ORF) of* amy*175 had 1722 bp encoding a protein of 573 amino acids residues. Multiple alignments indicated Amy175 had seven highly conserved sequences and the putative catalytic triad (Asp^244^, Glu^286^, and Asp^372^). It was the first identified member of GH13_36 subfamily which contained QPDLN in the CSR V. The recombinant enzyme (Amy175) was purified to homogeneity with a molecular mass of about 62 kDa on SDS-PAGE. It had a mixed enzyme specificity of *α*-amylase and *α*-glucosidase. Amy175 displayed highest activity at pH 8.0 and 25°C and exhibited extreme salt-resistance with the maximum activity at 1 M NaCl. Amy175 was strongly stimulated by Mg^2+^, Ni^2+^, K^+^, 1 mM Ca^2+^, 1 mM Ba^2+^, 1 mM Pb^2+^, 1 mM sodium dodecyl sulphate (SDS), and 10% dimethyl sulfoxide (DMSO) but was significantly inhibited by Cu^2+^, Mn^2+^, Hg^2+^, 10 mM *β*-mercaptoethanol (*β*-ME), and 10% Tween 80. Amy175 demonstrated excellent resistance towards all the tested commercial detergents, and wash performance analysis displayed that the addition of Amy175 improved the stain removal efficiency. This study demonstrated that Amy175 would be proposed as a novel *α*-amylase source for industrial application in the future.

## 1. Introduction

*α*-Amylases (E.C.3.2.1.1) are hydrolytic enzymes which can randomly cleave *α*-1,4-glycosidic linkages in starch molecules to generate gradually smaller polymers consisting of glucose units [1, 2]. As important industrial enzymes, amylases occupy about 25-30% of the world enzyme market [3, 4] and can be applied in numerous industries such as food, fermentation, detergent, paper, textile, pharmaceutical, and fine-chemical industries [5].

Most *α*-amylases belong to glycoside hydrolase family 13 (GH13). Among classification systems of glycoside hydrolases (GHs), the family GH13 forms the clan GH-H together with the families 70 and 77 [6]. The classification system of GH has been incorporated into the Carbohydrate-Active enZymes database (CAZy) [7]. As the largest GH family, the family GH13 consists of more than 30 different enzyme specificities and more than 55,500 sequences (http://www.cazy.org/GH13.html). It was officially divided into 35 subfamilies by the CAZy curators in 2006 [8]. The number of GH13 subfamilies has reached 42 currently and is still updating [9, 10]. Although the overall sequences of family GH13 members own very low identity, they possess 4-7 conserved sequence regions (CSRs) and a catalytic triad (Asp, Glu, and Asp) [11, 12].

Some subfamilies of GH13 enzymes are very closely related to each other [11], such as oligo-1,6-glucosidase, neopullulanase, and the intermediary group GH13_36 subfamily. The oligo-1,6-glucosidase subfamily includes mainly oligo-1,6-glucosidase, *α*-glucosidase, trehalose synthase, sucrose isomerase, trehalose-6-phosphate hydrolase, and dextran glucosidase, and the neopullulanase subfamily consists of cyclomaltodextrinase, maltogenic amylase, and neopullulanase [13], whereas the members of GH13_36 subfamily were reported to possess a mixture of enzyme specificity of *α*-amylase and some others from the two above-mentioned subfamilies [12]. There were efforts to define them based on specific features in their CSRs [12]. Originally oligo-1,6-glucosidase and neopullulanase subfamily were distinguished using specific sequence motif QPDLN and MPKLN in their CSR V, respectively. The subfamily GH13_36 was described with the sequence MPDLN discriminating the former two subfamilies [13]. Therefore CSR V can be used as a selection marker. The GH13_36 enzymes also possess other additional sequence features, such as an invariant tryptophan in the CSR VI and a tyrosine preceding the tripeptide “GEE” at the end of the CSR VII. Moreover, some GH13_36 members have furthermore a histidine at the end of the CSR II and a tryptophan (or other aromatic residues) in the CSR III [14]. These features can be used as reference information for subfamily assignment of GH13_36-like protein without additional biochemical characterization.

To improve the productivities of various industries, novel *α*-amylases with extreme properties such as activity at low/high-temperatures and salt-tolerance need be continuously sought for and applied in harsh industrial processing conditions. Cold-adapted enzymes that catalyze the reaction at low temperatures but lose activities by a moderate heating are highly beneficial to industries and biotechnology and have obtained increasing attentions in recent years [15–18]. For example, cold-adapted *α*-amylases can be added to detergents for cold washing to save the energy, reduce the wear, and protect the color of fabrics [19–21]. In baking processes, they can be used to shorten the dough fermentation time, quickly terminate the reaction of other enzymes, and improve the properties of the bread [19]. In addition, salt-tolerant enzymes have been widely used in detergent industry and bioremediation process. Many stains on fabrics have high salt concentration that requires salt-tolerant enzymes to remove them completely. Although several cold-adapted *α*-amylases [22–29] or salt-tolerant *α*-amylases [22, 24, 30–33] have been found, to the best of our knowledge, very few *α*-amylases possess both properties.

Antarctic is a unique ecosystem on earth, which is composed of a combination of extreme cold, high salt, and strong radiations. Organisms of Antarctic have evolved their specialized cold and high salt tolerant enzymes to adapt to and survive in this harsh environment. In this work, a novel *α*-amylase-producing strain* Pseudoalteromonas *sp. M175 (KU726544) was isolated and identified from Antarctic ice cover. A novel *α*-amylase gene of GH13_36 subfamily,* amy*175, isolated from* Pseudoalteromonas *sp. M175 was cloned and expressed in* E. coli*, and then the recombinant protein was purified and fully characterized. In addition, the primary application of Amy175 was tested as detergent additives.

## 2. Methods

### 2.1. Identification of Strain M175

Strain M175, the *α*-amylase producing strain, was isolated from Antarctic sea ice (68°30′E, 65°00′S) and was grown on 2216E medium (peptone 5.0 g, yeast extract 1.0 g, FePO_4_·2H_2_O 0.01 g, seawater 1 L, pH 7.5) at 15°C with shaking at 120 rpm. The organism was identified by physiological (Gram) and biochemical tests. Two primers were used for the amplification of 16S rRNA gene: 27F (5′-AGAGTTTGATCCTGGCTCA-3′) and 1492R (5′-GGTTACCTTGTTACGACTT-3′). Multiple sequence alignments were performed with the ClustalW software and a phylogenetic tree was constructed using MEGA 6.0 software.

### 2.2. Gene Cloning and Sequence Analysis

The genomic DNA of* Pseudoalteromonas* sp. M175 was prepared using Bacterial DNA Extraction Kit (Sangon Biotech, China). PCR primers were designed based on the sequence of the putative *α*-amylase gene of* Pseudoalteromonas haloplanktis* TAC125, whose genome sequence was released in GenBank (CR954246) by Médigue* et al.* [34], as follows: forward primer 5′-TGTTAATAGGCGCGGTGTC-3′ and reverse primer 5′-GGAGCTGTGCGTAGTAAC-3′. PCR was performed with the following conditions: 94°C, 5 min; 30 cycles of 94°C, 45 s; 55°C, 1 min; 72°C, 1 min and finally 72°C, 10 min. The PCR product was inserted into pGM-T and sequenced (Sangon Biotech, China).

The open reading frame (ORF) of* amy*175 was determined and translated to amino acid sequence using DNAMAN 5.2.2 software. The sequence analysis was performed by the BLAST program of NCBI (http://www.ncbi.nlm.nih.gov/blast). The prediction of signal peptide was conducted using the SignalP 4.1 Server (http://www.cbs.dtu.dk/services/SignalP). Molecular mass and theoretical p*I* were predicted by ProtParam tool (http://web.expasy.org/protparam/). The multiple sequence alignments were studied with 27 amylolytic enzymes from oligo-1,6-glucosidase (GH13 subfamilies 4, 16, 17, 18, 23, 29, 30, and 31), neopullulanase (GH13 subfamily 20), and GH13_36 subfamily. The neighbor-joining phylogenic tree was built by MEGA 6.0 with 1000 bootstrap replicates. The search for protein structure template during homology modeling was done using SWISS-MODEL (https://swissmodel.expasy.org/).

### 2.3. Expression and Purification of the Recombinant *α*-Amylase

The plasmid of pGM-*amy*175 was used as the template. To obtain the mature *α*-amylase, the *α*-amylase gene (*amy*175) was amplified using forward primer 5′-CGCGGATCCCCATCAACAAATACTAAC-3′ and reverse primer 5′-CCGCTCGAGCAGTGTGTTATTTAGTAACAAT-3′ (*BamH *I and* Xho *I sites underlined, respectively). The PCR product was gel purified, digested with* BamH *I and* Xho *I, and then ligated into the vector pET-28a(+) and transformed into competent* E. coli *DH5*α* cells. The recombinant plasmid pET-*amy*175 was transformed into competent* E. coli* BL21 (DE3) for protein expression and purification.

*E. coli* BL21 (DE3) cells with pET-*amy*175 were grown overnight at 37°C in LB medium containing 100 *μ*g/mL kanamycin. Subsequently, 1 mL culture was inoculated into 50 mL LB medium and cultured at 37°C to an optical density of 0.6 at 600 nm. Expression was induced with 0.3 mM isopropyl *β*-D-1-thiogalactopyranoside (IPTG) at 15°C for 12 h. Cells were collected by centrifugation at 7500 rpm for 20 min at 4°C and washed twice with the sterile water before being resuspended in the buffer (20 mM Tris-HCl pH 8.0, 0.5 M NaCl, 1 mM EDTA, 1 mg/mL lysozyme). The suspended solids were lysed using ultrasonic sonicator (Scientz, China) and then centrifuged at 12000 rpm for 15 min at 4°C. The supernatant was collected and applied to Ni-NTA resin affinity chromatography according to the manufacturer's instructions. Bound proteins were eluted with buffer containing 20 mM Tris-HCl, 0.5 M NaCl, 100 mM imidazole, pH 8.0.

The purity of the recombinant *α*-amylase was analyzed by 12% sodium dodecyl sulphate-polyacrylamide gel electrophoresis (SDS-PAGE) and the protein concentration was measured according to the Bradford method [35]. For zymogram study, the purified *α*-amylase was electrophoresed by SDS-free PAGE containing 1% (w/v) soluble starch. After electrophoresis, the gel was incubated in 50 mM Tris-HCl buffer (pH 8.0) for 1 h and then stained by iodine solution until a clear band appeared. The purified recombinant protein was analyzed by tandem mass spectrometry with MALDI-TOF (Bruker Daltonics, Germany) using the parameters described by Li et al. [36].

### 2.4. *α*-Amylase Activity Assay

The amylase activity was measured principally according to the Miller method [37], using the 3,5-dinitrosalicylic acid (DNS). In brief, 500 *μ*L of 50 mM Tris-HCl buffer (pH 8.0) containing 1% (w/v) soluble starch was maintained for 5 min at the desired temperature, and then 500 *μ*L of the purified enzyme was added to the buffer. After incubation for 10 min at 25°C, the reaction was stopped by the addition of 1 mL of DNS and the mixture was boiled for 5 min. The absorbance was measured at 540 nm using a UV/visible spectrophotometer (MAPADA, China). One unit of amylase activity was defined as the amount of enzyme that released 1 mg/mL maltose equivalent to reducing sugars per minute under the assay conditions.

### 2.5. Effects of Temperature and pH on Enzymatic Activity and Stability

The optimum temperature of the enzyme against the soluble starch was investigated by incubating the reaction mixture at different temperatures (0-60°C) in 50 mM Tris-HCl buffer (pH 8.0). To study the thermostability, the purified recombinant *α*-amylase was held at 30, 40, and 50°C for different time periods and the residual activity was measured as described above.

The optimum pH of the enzyme was analyzed by incubating the reaction mixture in the range of pH 5.0-11.0 at 25°C using the following buffers (50 mM): Na_2_HPO_4_-citric acid (pH 5.0-7.0), Tris-HCl (pH 7.0-9.0), and glycine-NaOH (pH 9.0-11.0). The pH stability test was performed by incubating the enzyme in the different buffers at 25°C for 1 h. The remaining activity was measured under standard conditions as described above.

### 2.6. Effects of NaCl on Enzymatic Activity and Stability

The effect of NaCl on enzyme activity was tested in the range of 0-5 M at 25°C in 50 mM This-HCl buffer (pH 8.0). The effects of NaCl on enzyme stability were studied by preincubating the enzyme in 50 mM This-HCl buffer (pH 8.0) with 1 M NaCl and without NaCl, respectively, at 25°C for 0-120 min. The remaining activity was measured under standard conditions as described above. The enzyme activity at 0 min was defined as 100%.

### 2.7. Effects of Metal Ions and Chemical Reagents

The effects of different metal ions, sodium dodecyl sulphate (SDS), EDTA, dithiothreitol (DTT), *β*-mercaptoethanol (*β*-ME), and urea on enzyme activity were determined at the final concentration of 1 mM and 10 mM. The effects of Tween 80, Triton X-100, and dimethyl sulfoxide (DMSO) on enzyme activity were tested at 1% and 10% concentrations. The enzyme activity was measured in 50 mM This-HCl buffer (pH 8.0) containing 1% (w/v) soluble starch at 25°C.

### 2.8. Determination of Kinetic Parameters

The kinetic parameters were determined by incubating the enzyme with different concentrations of soluble starch ranging from 0.125% to 2% (w/v) in 50 mM Tris-HCl buffer (pH 8.0) under standard conditions. V_max_ and K_m_ values were calculated from Lineweaver-Burk plot.

### 2.9. Substrate Specificity and Products Analysis by Thin Layer Chromatography (TLC)

The activities on *α*-, *β*-, and r-cyclodextrin (CD), soluble starch, glycogen, amylose, amylopectin, pullulan, and several raw starches such as potato starch, corn starch, wheat starch, and pea starch were detected using DNS method at 1% (w/v) concentration during the enzyme assay. The enzyme activity for soluble starch was defined as 100%. The activity on several oligosaccharides (maltose, maltotriose, maltotetraose, sucrose, isomaltose, trehalose, raffinose, panose, and melibiose) at 1% (w/v) concentration was determined by assaying the release of glucose at 25°C for various periods with a glucose assay kit (Sigma Diagnostics no. 510). Additionally, degradation of* p*-nitrophenyl-*α*-D-glucopyranoside (*p*NPG) was evaluated by measuring the release of* p*-nitrophenol at 410 nm after the reaction was terminated by adding an equal volume of 1 M Na_2_CO_3_.

To analyze the end hydrolysis product, the recombinant enzyme was incubated with 1% (w/v) soluble starch, maltose, maltotriose, and maltotetraose, in 50 mM Tris-HCl buffer (pH 8.0) at 25°C for various periods and 10 *μ*L of the reaction mixtures was submitted to TLC on 0.25-mm silica gel plates (GF254, 20 cm × 20 cm). Ethyl acetate-acetic acid-methanol-water (12:3:3:2, v/v) was used as the mobile phase, and the products were detected by exposing the plate to 2.0% aniline-2.0% diphenylamine-10.0% phosphate in acetone, followed by drying at 80°C for 15 min.

### 2.10. Compatibility Test with Various Commercial Detergents

To confirm the potential of the recombinant enzyme as a laundry detergent additive, its compatibility and stability against various Chinese liquid commercial detergents, such as Liby®, Walch®, OMO®, Blue Moon®, Diaopai®, Tide®, Keon®, Ariel®, and Chaoneng®, were checked. All detergents at 1% (v/v) concentration were heated at 100°C for 60 min to inactivate the endogenous enzyme prior to addition of enzyme preparation. The purified recombinant enzyme was incubated with individual detergent solution for 1 h at 25°C, respectively, and then the residual activity was determined. The reaction solution without detergents was considered as the control.

### 2.11. Wash Performance Analysis

Application of the recombinant enzyme as a detergent additive was evaluated on white cotton fabrics (4×4 cm^2^) stained with chocolate and tomato sauce. To determine stain removal efficiency, each piece of stained cloth was soaked separately in any one of the following flasks containing (a) distilled water (25 mL), (b) distilled water (20 mL) and 5 mL Tide® detergent (1%), and (c) distilled water (20 mL) and 5 mL Tide® detergent (1%) containing 12.6 U/mL of purified recombinant enzyme. The above flasks were kept at room temperature (25°C) for 1 h. After treatment, cloth pieces were rinsed with distilled water and dried. The wash performance of the purified enzyme was judged by visual examination.

### 2.12. Data Analysis

All data are expressed as mean value ± standard errors of at least triplicate determinations. The figures were drawn using Origin 8.0 software (OriginLab Corporation, USA) and statistical difference significance was analyzed using the SPSS 16.0 (*SPSS*, Chicago, IL) package for Windows.

## 3. Results

### 3.1. Identification of Strain M175

Strain M175, the *α*-amylase producing strain, isolated from Antarctic sea ice is aerobic, Gram-negative. It forms smooth and bright yellow colonies on nutrient medium. Growth occurs at 0°C-40°C (optimal temperature, 15°C) and pH 3.0-11.0 (optimal pH, 8.0). NaCl is not essential for growth, but growth is enhanced in the presence of NaCl (optimal NaCl, 6%, tolerated up to 12%). It is positive for catalase, indol production, gelatin hydrolysis, and Voges-Proskaeur (VP) test. However, Methyl-Red (MR) test and nitrate reduction are all negative. The result of Oxidation/Fermentation (O/F) Test is oxidation. It can utilize the following substrates as carbon sources: D-glucose, D-lactose, D-maltose, D-xylose, sucrose, and starch (Table 1).

**Figure 1:**
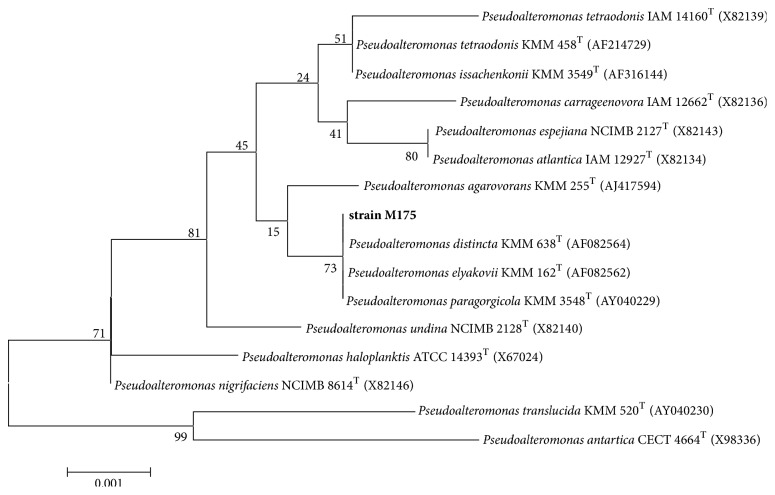
Neighbor-joining tree based on the 16S rDNA sequences of strain M175 and other* Pseudoalteromonas *species. Bootstrap values are based on 1000 replicates. All sequences were retrieved from the GenBank database.

The 16S rDNA analysis indicated that it was closely related to the genus* Pseudoalteromonas* with the highest levels of similarity (99%) to* Pseudoalteromonas distincta* KMM 638^T^ (AF082564),* Pseudoalteromonas elyakovii* KMM 162^T^ (AF082562), and* Pseudoalteromonas paragorgicola* KMM 3548^T^ (AY040229) (Figure 1). Based on the results including physiological, biochemical, and 16S rDNA alignment analyses, the strain was identified to be a member of* Pseudoalteromonas*, as* Pseudoalteromonas* sp. M175. The sequence has been submitted in GenBank with accession number KU726544.

### 3.2. Gene Cloning and Sequence Analysis

A DNA sequence (namely,* amy*175) of 1722 bp (GenBank accession number KC306394) was successfully cloned from Antarctic sea ice bacterium* Pseudoalteromonas *sp. M175.* amy*175 encodes a protein of 573 amino acids, which contains a predicted N-terminal signal peptide comprising 23 amino acids and a mature *α*-amylase (Amy175) with a calculated molecular weight of 62.4 kDa and p*I* of 4.9. Blastp homology search against the NCBI nonredundant protein database showed that Amy175 shared the high identity with putative *α*-amylase of* Pseudoalteromonas nigrifaciens* KMM661 (99%),* Pseudoalteromonas haloplanktis* TAC125 (99%),* Pseudoalteromonas atlantica* (77%),* Pseudoalteromonas undina* (77%),* Rheinheimera perlucida* (74%), and* Rheinheimera baltica *(74%). However, *α*-amylases from bacterium KMM661 and TAC125 were reported only in NCBI database with accession numbers of WP_089368202 and WP_041454408, respectively, and no further research on their cloning and properties. Furthermore, Amy175 shared the very low sequence identity with the studied *α*-amylases, such as 30%, 26%, 26%, 26%, 24% 22%, and 22% identity with *α*-amylase from* Escherichia coli*,* Pseudoalteromonas haloplanktis *TAB23,* Pseudoalteromonas arctica* GS230,* Bacillus cereus*,* Exiguobacterium *sp. SH3,* Thermobifida fusca*, and* Lipomyces starkey*i, respectively. Therefore, Amy175 is a novel *α*-amylase.

**Figure 2:**
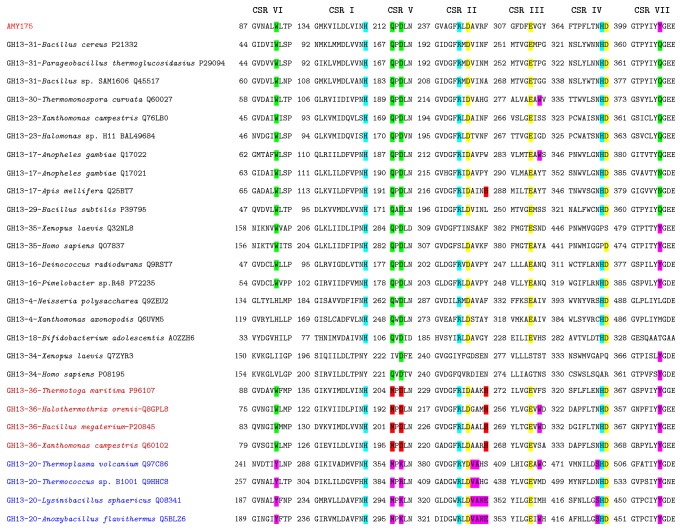
The seven conserved regions of Amy175 with other known amylolytic enzymes from oligo-1,6-glucosidase (GH13 subfamilies 4, 16, 17, 18, 23, 29, 30, and 31), neopullulanase (GH13 subfamily 20), and GH13_36 subfamily. The characteristic sequence is highlighted as follows: entire family GH13-specific-blue; catalytic triad-yellow; oligo-1,6-glucosidase-specific-green; neopullulanase-specific-purple; intermediary GH13_36-specific-red. The name of an enzyme is composed of the GH13 subfamily number, source (organism), and the UniProt accession number.

**Figure 3:**
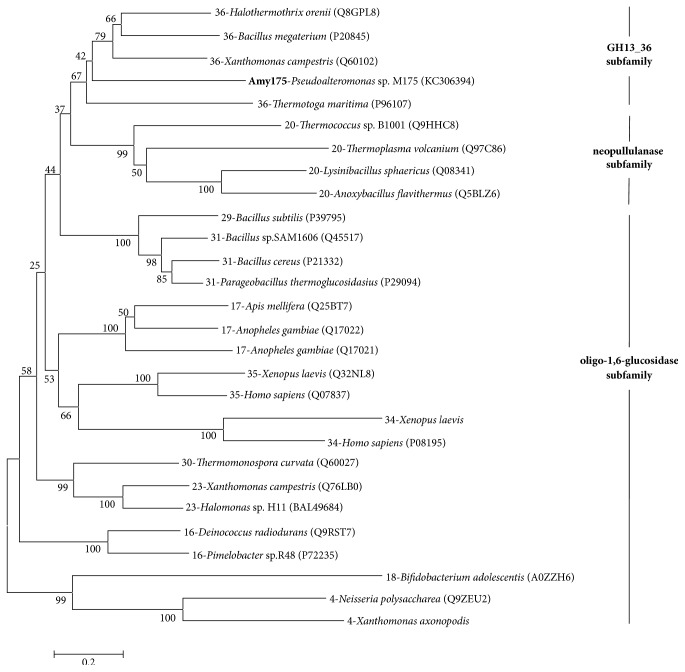
Phylogenic tree analysis of Amy175. The tree based on amino acid sequences of the Amy175 and the amylolytic enzymes from oligo-1,6-glucosidase, neopullulanase, and GH13_36 subfamily was constructed by the MEGA6 software with the neighbor-joining method and 1000 bootstrap replicates. All sequences were retrieved from the UniProt database.

The result of multiple sequence alignment indicated that Amy175 had seven highly conserved regions and the putative catalytic triad (Asp^244^, Glu^286^, and Asp^372^) which are the common characteristics of GH13 members [11] (Figure 2). It contained QPDLN in the CSR V characteristic for oligo-1,6-glucosidase subfamily. Similar to GH13_36, it had a tryptophan in the CSR VI and a tyrosine preceding the tripeptide “GEE” in its CSR VII. The tyrosine also often existed in the neopullulanase subfamily members [13]. But there was a phenylalanine rather than a histidine at the end of the CSR II, and a glycine not an aromatic residue in the CSR III. To analyze the evolutionary relationships of Amy175, 27 amylolytic enzymes that have already been biochemically characterized from these three subfamilies were selected and phylogenic tree was generated based on the neighbor-joining method by MEGA 6.0 software (Figure 3). The result revealed that Amy175 showed a closer relationship with GH13_36 members.

### 3.3. Expression and Purification of Amy175

**Figure 4:**
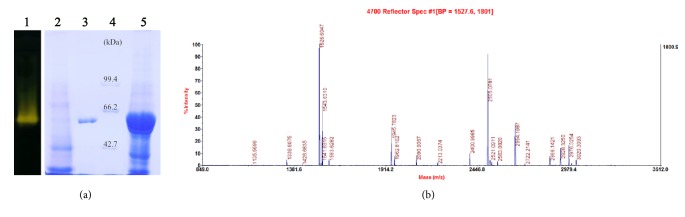
Expression of* amy*175 in* E. coli* BL21 (DE3). (a) Native-PAGE and SDS-PAGE analysis of the purified Amy175. Lane 1, native-PAGE of purified enzyme; Lane 2, noninduced protein extracts of* E. coli*; Lane 3, purified recombinant protein of Amy175; Lane 4, protein molecular mass marker; Lane 5,* E. coli* extracts with Amy175 expression induced. (b) MALDI-TOF-MS analysis of the purified recombinant Amy175.

The* amy*175 gene was successfully expressed in* E. coli* BL21 (DE3) as a His-tagged fusion protein (Figure 4(a)). A clear target band was found in the induced cells (lane 5), but not in the noninduced cells (lane 2) by SDS-PAGE analysis. The purified recombinant enzyme using Ni-NTA affinity chromatography showed a single band (lane 3) with approximate molecular mass of 62 kDa and the recombinant protein was confirmed by native-PAGE with *α*-amylase activity staining (lane 1). The enzyme was purified with the protein concentration of 385.1 *μ*g/mL and a specific activity of 337.9 U/mg that was higher than that of *α*-amylases, e.g.,* Pseudoalteromonas arctica* GS230 (25.5 U/mg) [23],* Zunongwangia profunda* (270.6 U/mg) [24], and* Pseudoalteromonas *sp. MY-1 (44.4 U/mg) [27], but lower than that of *α*--amylase from* Geomyces pannorum* (9.72×10^3^ U/mg) [38]. Additionally, the purified enzyme obtained on SDS-PAGE was excised and was submitted for MALDI-TOF-MS analysis. The peptide mass fingerprint (Figure 4(b)) was matched with the available bacteria database and revealed significant matches against *α*-amylases from* Pseudoalteromonas nigrifaciens* KMM661 and* Pseudoalteromonas haloplanktis* TAC125 with scores of 186 and 131, respectively.

### 3.4. The Effects of Temperature and pH on Enzymatic Activity and Stability

**Figure 5:**
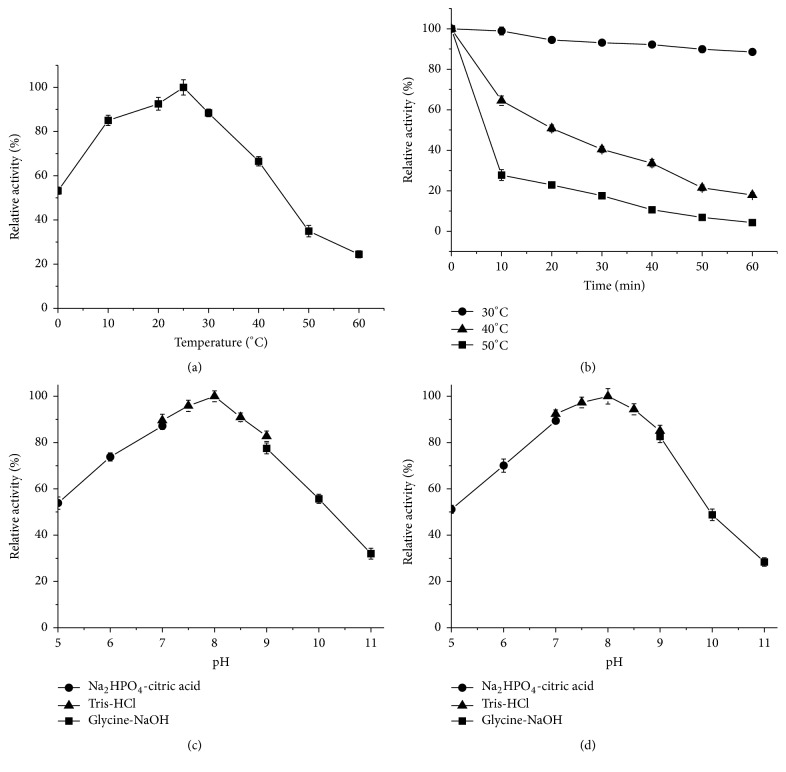
The effects of temperature and pH on the activity and stability of Amy175. (a) The effect of temperature on the activity of Amy175 was determined at the range of 0-60°C. The activity at 25°C was defined as 100%. (b) The thermostability of Amy175 was analyzed by preincubating the enzyme at 30, 40, and 50°C for the given times. The activity at 0 min was defined as 100%. (c) The effect of pH on the activity of Amy175 was measured at different pH (5.0-11.0), with the activity at pH 8.0 as 100%. (d) The pH stability of Amy175 was analyzed by preincubating enzyme at different pH (5.0-11.0) at 25°C for 1 h and the residual activity was determined at pH 8.0. The residual activity after preincubation at pH 8.0 was defined as 100%. Error bars represent the standard deviation of three independent measurements.

The influence of temperature on the activity of Amy175 was measured in the range of 0-60°C (Figure 5(a)). The result showed that Amy175 exhibited high activity at low temperature with maximum activity observed at 25°C and retained about 53.2% activity at 0°C, but its activity decreased sharply above 50°C. According to temperature-stability profile depicted in Figure 5(b), Amy175 was highly stable at 30°C and could keep about 88.6% activity after 60 min incubation, whereas at 40°C and 50°C it showed poor stability, losing about 35.5% and 72.3% activity, respectively, after 10 min incubation.

The effect of pH on the activity of Amy175 was tested in the range of pH 5.0-11.0 at 25°C (Figure 5(c)). The maximum activity was observed at pH 8.0 and more than 73.0% of the maximum activity could be still retained within pH 6.0-9.0. The pH stability results (Figure 5(d)) revealed that the enzyme was relatively stable and could remain more than 80.0% activity in a pH range of 7.0-9.0 for 1 h when assayed at 25°C, while the activity sharply decreased after preincubated at pH 10.0.

### 3.5. The Effects of NaCl Concentration on Enzyme Activity and Stability

**Figure 6:**
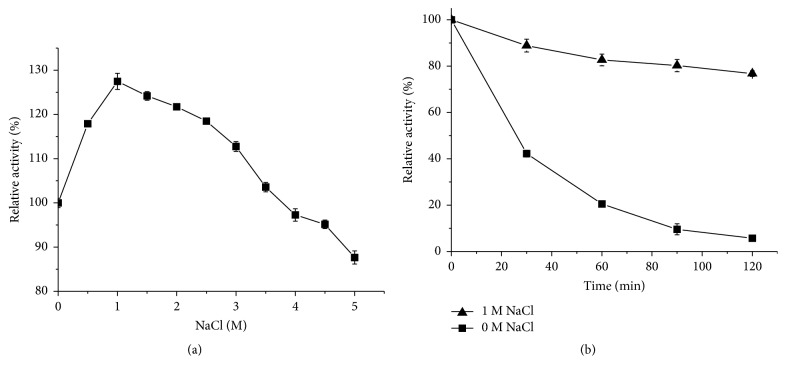
The effects of NaCl on the activity and stability of Amy175. (a) The effect of NaCl on the activity of Amy175 was measured at 25°C in 50 mM Tris-HCl buffer (pH 8.0) containing 0-5 M NaCl. The activity without NaCl was defined as 100%. (b) The effect of NaCl on stability was studied by preincubating enzymes at 25°C in 50 mM Tris-HCl buffer (pH 8.0) containing 0 or 1 M NaCl for the given times. The activity of Amy175 at 0 min was defined as 100%. Error bars represent the standard deviation of three independent measurements.

The effect of NaCl on the activity of Amy175 was analyzed in the range of 0-5 M (Figure 6(a)). The enzyme exhibited the highest activity in the presence of 1 M NaCl, showing 127.5% of original activity without NaCl. It could display 87.7% of original activity even at 5 M NaCl, suggesting that Amy175 was halotolerant. In addition, the stability of enzyme was detected under the presence of 0 M and 1 M NaCl for 2 h. As shown in Figure 6(b), the presence of NaCl could improve the stability of Amy175. The activity of Amy175 was rapidly lost when preincubated without NaCl at 40°C for 120 min. However, the residual activity was increased to 82.7% and 76.7%, respectively, after preincubated with 1 M NaCl for 60 min and 120 min.

### 3.6. The Effects of Metal Ions and Chemical Reagents on Enzymatic Activity

Of metal ions tested (Table 2), Mg^2+^, Ni^2+^, and K^+^ at both tested concentrations stimulated the activity of Amy175, and the highest activity reached 171.2% in the presence of 10 mM Mg^2+^. Interestingly, Amy175 was increased by 1 mM Ca^2+^ (121.3%), Ba^2+^ (119.4%), and Pb^2+^ (112.3%) but was decreased by 10 mM Ca^2+^ (79.1%), Ba^2+^ (78.9%), and Pb^2+^ (73.1%). Furthermore, Cu^2+^, Mn^2+^, and Hg^2+^ were the strong inhibitors, and partial inhibition of Amy175 was observed in the presence of Al^3+^, Fe^2+^, Fe^3+^, and Cd^2+^.

Effects of several chemical reagents on Amy175 were assessed (Table 3). *β*-ME (10 mM) and Tween 80 (10%) strongly inhibited the enzyme activity by 31.9% and 48.4%, while DMSO (10%) and SDS (1 mM) increased the activity by 141.9% and 118.8%, and the enzyme activities were retained more than 69.0% in the presence of EDTA, DTT, Urea, and Triton X-100 at both tested concentrations.

### 3.7. Kinetics Parameters

Kinetic studies of Amy175 were determined under standard conditions using different concentrations of soluble starch (0.125%-2%) as substrate. As obtained from the Lineweaver-Burk plot, the K_m_ and V_max_ values were 2.53 mg/mL and 0.125 mg/mL/min, respectively. The K_m_ value for Amy175 was lower than that of the *α*-amylases from* Pseudoalteromonas arctica* GS230 (7.28 mg/mL) [23] and* Zunongwangia profunda *(2.74 mg/mL) [24], but higher than that of *α*-amylase from* Exiguobacterium *sp. SH3 (2.29 mg/mL) [25].

### 3.8. Analysis of Substrate Specificity and Hydrolysis Products

**Figure 7:**
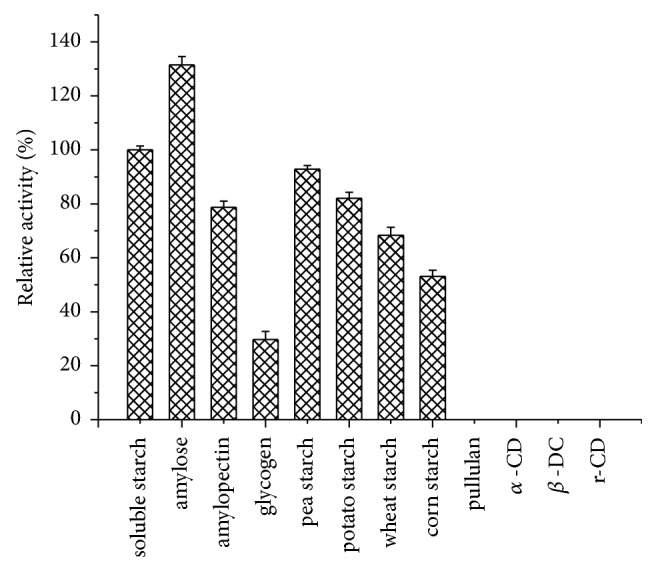
Substrate specificity of purified Amy175. Error bars represent the standard deviation of three independent measurements.

Substrate specificity of *α*-amylase varies with the source of microorganism [39]. In Figure 7, Amy175 displayed the highest specificity towards amylose (131.4%), which is a linear glucose polymer connected by *α*-1,4 glycosidic bonds, followed by soluble starch (100%), while amylopectin (78.7%) and glycogen (29.7%) had much lower rate of hydrolysis due to more branches connected by *α*-1,6 glycosidic bonds in them. Moreover, Amy175 could not hydrolyze pullulan, *α*-, *β*-, and r-CD. Therefore, it is suggested that Amy175 prefers *α*-(1,4) linkage cleaving. Amy175 displayed the highest rate of hydrolysis towards pea starch (92.8%), followed by potato starch (82.1%), wheat starch (68.3%), and corn starch (53.1%). Amy175 showed different hydrolysis abilities towards various starches, due to the difference in particle size and shape, the ratio of amylose and amylopectin, and structure of the amylose and amylopectin molecules [40]. The result of oligosaccharides hydrolysis showed that the enzyme could not degrade other oligosaccharides except maltooligosaccharides and no glucose was observed even after incubation for 48 h. In addition, the enzyme was not able to hydrolyze* p*NPG. It is further indicated that the enzyme only cleaved *α*-(1,4) linkage.

**Figure 8:**
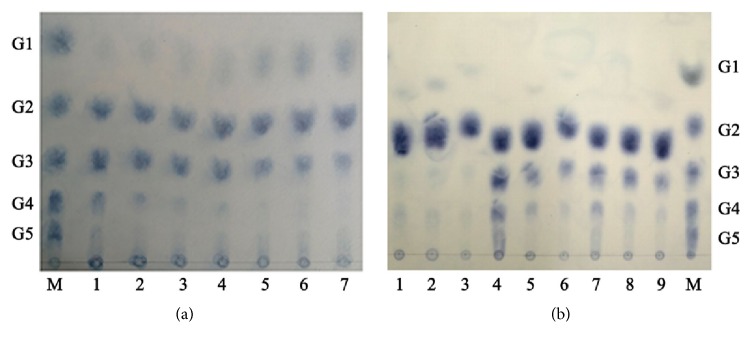
TLC analysis of product formation during degradation of soluble starch and maltooligosaccharides. Lane M, maltooligosaccharide standards (G1 glucose, G2 maltose, G3 maltotriose, G4 maltotetraose, and G5 maltopentaose). (a) The degradation products of soluble starch. Lane 1 to Lane 7: the end products after incubation of Amy175 with soluble starch at 25°C for 15 min, 1 h, 3 h, 6 h, 12 h, 24 h, and 48 h, respectively. (b) The degradation products of maltooligosaccharides. Lanes 1-3: the end products of G2 for 1 h, 24 h, and 48 h; Lanes 4-6: the end products of G3 for 1 h, 24 h, and 48 h; Lanes 7-9: the end products of G4 for 1 h, 24 h, and 48 h, respectively.

The hydrolysis products of soluble starch analyzed by TLC for various periods were shown in Figure 8(a). At the early stage of hydrolysis, soluble starch was hydrolyzed into maltose (G2), maltotriose (G3), maltotetraose (G4), and a small amount of higher molecular weight oligosaccharides. As the reaction proceeded, glucose (G1) gradually increased; however, G2 was still the main hydrolysis product after incubation for 48 h. These hydrolysis patterns suggest that Amy175 mainly cleave *α*-1,4-glycosidic linkage in the interior of the starch, having great demands in the food and starch industry, such as bio-ethanol production and baking industry.

To determine whether Amy175 could perform transglycosylation, the hydrolysis products of maltooligosaccharides were analyzed by TLC (Figure 8(b)). Amy175 released a small amount of glucose from maltose at 1 h. When maltotriose and maltotetraose were degraded, Amy175 also produced oligosaccharides that were one-glucose unit smaller than the substrates. It is noteworthy that the oligosaccharides that were larger than the original substrates were produced by Amy175, especially when maltotriose was hydrolyzed for 1 h. The results suggested that Amy175 possessed transglycosylation activity.

### 3.9. Detergency Characteristics

**Figure 9:**
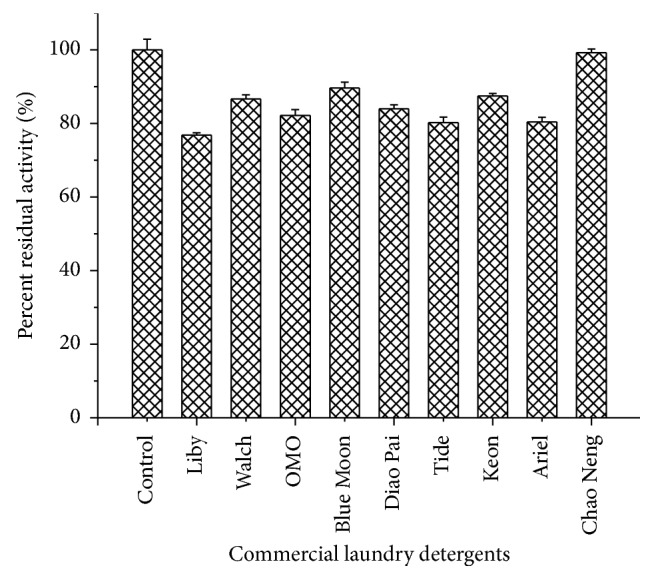
Detergent compatibility study of the purified Amy175. Error bars represent the standard deviation of three independent measurements.

To confirm the application of Amy175 in detergent formulations, its stability was investigated in presence of various commercial detergents (Figure 9). The result revealed that Amy175 exhibited extreme stability with all the tested commercial laundry detergents and more than 76.9% residual amylase activity was retained. Chaoneng® was most compatible, since after 1 h of incubation at 25°C, 99.3% of its initial activity remained.

### 3.10. Wash Performance Analysis

**Figure 10:**
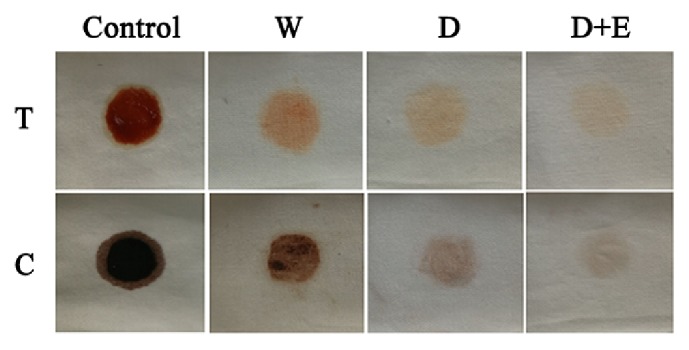
Wash performance analysis of Amy175, the amylase from* Pseudoalteromonas *sp. M175. Washing of stained cotton fabrics was kept with three different sets of washing solutions (W, D, D + E) at room temperature for 1 h. Rows: T: clothes stained with tomato sauce; Lane C: clothes stained with chocolate. Control: stained clothes before wash; W: washed with distilled water only; D: washed with detergent only; D + E: washed with detergent added with Amy175.

Stained cotton fabrics washed by three different sets of washing solutions revealed that a combination of the purified *α*-amylase and detergent (Tide®) together resulted in best washing (Figure 10). Although the detergent alone showed fainted washing effect on chocolate and tomato sauce spots (starch rich), addition of Amy175 (with 12.6 U/mL of activity) improved the stain removal efficiency. This result indicated that Amy175 could be employed as potential laundry detergent additive.

## 4. Discussion

In this study, a novel cold-adapted, salt-tolerant, and detergent-stable *α*-amylase gene (*amy*175) from Antarctic sea ice bacterium* Pseudoalteromonas *sp. M175 was cloned, expressed, and characterized.

Amy175 had seven highly conserved regions and the putative catalytic triad. It contained QPDLN in the CSR V features for oligo-1,6-glucosidase subfamily. Although CSR V was often used as a selection marker, CSR V alone could not be enough to identify a GH13 protein's subfamily. Several GH13 amylolytic enzymes and proteins with MPDLN were assigned to the oligo-1,6-glucosidase subfamily, such as the proteins from* Lactobacillus sakei* (UniProt: Q38WC9),* Grosmannia clavigera* (UniProt: F0XH23), and* Sordaria macrospora* (UniProt: D1ZB31) [14]. Furthermore, the mammalian amino acid transporter also contained the oligo-1,6-glucosidase-type of QPDLN [12]. Of course, no QPDLN-containing proteins have been assigned to the GH13_36 subfamily so far. Amy175 behaved like the member of the GH13_36 subfamily in the CSR VI and CSR VII. However, unlike most GH13_36 members, it did not have a histidine at the end of the CSR II, which was similar to the intermediary GH13_36 *α*-amylase from* Bacillus clarkii* (UniProt: B9A1J7). Moreover, it did not contain an aromatic residue in the CSR III, and several GH13_36 enzymes from* Xanthomonas campestris* (UniProt: Q60102),* Thermotoga maritima* (UniProt: P96107), and uncultured bacterium (UniProt: Q6TXT5) also had similar characteristics. The evolutionary relationships among 27 amylolytic enzymes from GH13 family showed that Amy175 possessed a closer relationship with GH13_36 subfamily.

For most GH13_36 members, the intermediary GH13_36 *α*-amylase AmyA (PDB: 1WZA) from* Halothermothrix orenii* [40] was recognized as the best template, while the best templates for a few GH13_36 members were also identified from oligo-1,6-glucosidase subfamily [14]. The best structural template of Amy175 for homology modeling showed by SWISS-MODEL was a trehalose synthase (PDB: 5H2T) from the oligo-1,6-glucosidase subfamily. Its coverage value (0.87) was higher than AmyA with coverage value of 0.74. However, its sequence identity with Amy175 was only 31.59, lower than 41.37 of AmyA. On the other hand, this trehalose synthase was described as a homotetramer, while the result of native-PAGE indicated that Amy175 should be a monomer. Therefore, *α*-amylase AmyA was more suitable as the template, and Amy175 was similar to GH13_36 *α*-amylase structurally.

The result that Amy175 degraded the soluble starch to several maltooligosaccharides suggests that Amy175 mainly hydrolyze starch internally (Figure 8(a)). It could not hydrolyze* p*NPG and other oligosaccharides except maltooligosaccharides, which demonstrated Amy175 could not cleave *α*-1,6-, *α*-1,1, and *α*-1,2 bond, but could catalyze the hydrolysis of terminal *α*-1,4-glucosidic linkages. Moreover, Amy175 possessed transglycosylation activity. These hydrolysis patterns suggest that Amy175 have not only *α*-amylase activity, but also *α*-glucosidase activity from oligo-1,6-glucosidase subfamily. Thus, Amy175 should be a novel member of *α*-amylase GH13_36 subfamily, which often possess a mixed enzyme specificity of *α*-amylase, oligo-1,6-glucosidase subfamily, and neopullulanase subfamily [12].

To date, several cold-adapted *α*-amylases which have been cloned and expressed were listed in Table 4. Amy175 showed lower optimal temperature (25°C) than other cold-adapted *α*-amylases, with exceptions of AHA (25°C) from the psychrophilic bacterium* Alteromonas haloplanktis *and Amy_13C6_ (10-15°C) from a metagenomic library. Furthermore, Amy175 could still keep 53.2% maximum activity at 0°C, while the activities retained at 0°C by AmyZ from* Zunongwangia profunda *[24], ParAmy from* Pseudoalteromonas arctica* GS230 [23], and AHA from* Alteromonas haloplanktis* [22] were 39.0%, 34.5%, and 20.0%, respectively. However, the activity of Amy175 decreased sharply above 50°C and only about 64.5% and 27.7% activity were retained, respectively, after 10 min incubation at 40°C and 50°C, indicating relatively low thermostability, which is the typical characteristic of cold-adapted enzymes and makes inactivating the enzyme in special applications more easy and rapid [41].

Cold-adapted enzymes can carry out their functions at very low temperature because of their flexible structures [22]. Cold-adapted enzymes usually possess less arginine residues or a lower arginine/(arginine and lysine) ratio than the mesophilic and thermophilic enzymes [42]. Arginine is famous as a stabilizing residue and can reduce the flexibility by forming hydrogen bands and salt bridges with the guanidinium group [42, 43]. Analysis of the amino acid sequence showed that Amy175 has less arginine residues and a lower arginine/(arginine and lysine) ratio than some other cold-active, mesophilic, or thermophilic *α*-amylases, which may partly explain its cold activity (Table 5).

Another noticeable characteristic of Amy175 was its salt-tolerance. Amy175 exhibited the activity in a wide range of 0-5 M NaCl with the highest activity in the presence of 1 M NaCl (127.5% of original activity) and 87.7% activity was retained even at 5 M NaCl (Figure 6(a)). However, Amy175 is a salt-tolerant enzyme, but not a halophilic enzyme, because halophilic enzymes would be unstable and rapidly lose the activities in the absence of the salt [46], such as the halophilic *α*-amylases produced by* Marinobacter* sp. EMB8 [30] and* Halorubrum xinjiangense* [31]. In addition, the stability of Amy175 could be dramatically improved by NaCl (Figure 6(b)), which was similar to that of the *α*-amylases from marine bacterium* Zunongwangia profunda* [24, 32] and halophile bacterium* Halothermothrix orenii *[33]. Some researches showed that the hydrophobic interactions of enzyme core structures were possibly enhanced by salting-out effect under high salinity and made enzymes more compact and stable [26], which might be helpful to enhance the stability of these enzymes.

Halophilic and salt-tolerant *α*-amylases have more acidic amino acids residues (Asp and Glu) than basic amino acids residues (Lys and Arg) [47]. An abundance of acidic amino acids produces a negative surface potential, promoting the formation of the hydrated salt ions network that reduces the tendency of aggregation and keeps the enzyme activity and stability under high salinity [48–50]. The proportion of acidic amino acid excess of Amy175 is 5.0%, which was higher than that of AHA (4.2%) from* Alteromonas haloplanktis *and Amy13A (4.8%) isolated from a pilot-plant biogas reactor, but lower than that of AmyZ (5.7%) from* Zunongwangia profunda*, AmyH (10.4%) from the halophilic archaeon* Haloarcula hispanica, *and *α*-amylase^A^ (18.9%) from the archaebacterium* Natronococcus* sp. strain Ah-36 (Table 6). In addition, Amy175 was predicted to be an extracellular enzyme with N-terminal signal peptide of 23 amino acids. Qin et al. [24] found that the extracellular proteins were more salt-tolerant than intracellular proteins by studying the proteins from* Zunongwangia profunda*.

Additionally, effects of various metal ions and chemical reagents on enzyme activity were studied (Tables 2 and 3). Cu^2+^, Mn^2+^, and Hg^2+^ strongly decreased activity of Amy175. These metal ions may inhibit the enzyme activity by either binding to catalytic residues or replacing the required metal ions [53]. The *α*-amylases from* Exiguobacterium* sp. DAU5 [54] and* Eisenia foetida *[55] were also inhibited by Cu^2+^ and Hg^2+^, whereas some *α*-amylases, i.e., *α*-amylase from* Bacillus licheniformis* AT70, were activated by Cu^2+^ [56]. Nies reported that Hg^2+^ could bind to thiol groups in the *α*-amylase structure to reduce its activity [57]. Amy175 was also inhibited by *β*-ME (10 mM) and Tween 80 (10%). In contrast, the *α*-amylase of* Arthrobacter agilis *PAMC 27388 was enhanced by *β*-ME [58]. Interestingly, DMSO (10%) and SDS (1 mM) increased the activity of Amy175. The enzyme resistance towards SDS is a good characteristic, particularly in detergent industry, and SDS-stable amylases have been rarely reported [59]. Furthermore, Amy175 exhibited the tolerance to other chemical reagents and could keep more than 69.0% of the original activity. Despite loss of activity of the most *α*-amylases with EDTA [60, 61], Amy175 had completely retained its original activity with 1 mM EDTA and kept 88.9% of its original activity even with 10 mM EDTA, which was similar to the result on* Bacillus* KSM-K38 *α*-amylase [62]. In addition, the activity of the *α*-amylase from* Bacillus* licheniformis AT70 increased about 145% in the presence of EDTA [56]. Arikan reported that some alkaline amylases were unaffected by chelator EDTA [63]. It is probable that some metal ions can activate Amy175, but they are not essential for the catalytic reaction process.

Amy175 demonstrated not only good tolerance towards some chemical reagents, but also excellent stability against all the tested commercial detergents. In contrast, the *α*-amylase from marine* Streptomyces* sp. D1 retained only 35-70% of its original activity in the presence of commercial detergents [64]. Some components of commercial detergents, for example, anionic surfactants, water softening builders, and stabilizers, may have inhibitory effect on the *α*-amylase activity, while some other ingredients such as ethoxylated surfactants and nonionic copolymeric builders may stimulate the *α*-amylase activity [65]. Therefore, the residual amylase activity is the result of combining effects of different ingredients in the detergents.

Furthermore, the addition of Amy175 led to better stain removal from cotton fabrics than that of detergent and water alone. The amylases can help to enhance wash performance by effectively breaking down starch rich stains, protect the environment due to the biodegradability of enzymes, and make laundry detergent more sustainable [20]. All above results suggested that Amy175 could be added to laundry detergent formulations, for enhancing the ability of detergents to clean clothes in cold water.

## 5. Conclusion

In summary, a novel *α*-amylase-producing strain* Pseudoalteromonas *sp. M175 (KU726544) was isolated from Antarctic ice cover and identified by physiological, biochemical, and 16S rDNA alignment analyses. A novel *α*-amylase, Amy175, from* Pseudoalteromonas *sp. M175, was expressed and purified. To our knowledge, it was the first identified member of GH13_36 subfamily containing QPDLN in CSR V. It was found to have a mixed enzyme specificity of *α*-amylase and *α*-glucosidase and possess several remarkable extreme-condition tolerance characteristics: cold-active, salt-tolerant, and relatively stable in various detergents. Such distinctive characteristics suggest that this enzyme has potential industrial applications in which low temperature-processing is required, and/or the high concentration of salts is present, and/or the residue solvents remained from prior treatments. With the global trend of using low temperature processing to save energy, cold-active amylases such as Amy175 would be a promising enzyme candidate to be used for industries such as food, detergent, and textile.

## Figures and Tables

**Table 1:** Biochemical, morphological, and physiological characteristics of strain M175.

Experiments	Results
Gram	Gram-negative
	aerobic
Growth range	0-40°C, pH 3.0-11.0, 0-12% NaCl
Optimal temperature	15°C
Optimal pH	pH 8.0
Optimal NaCl	6%
Catalase	+
Indol production	+
Gelatin hydrolysis	+
VP	+
MR	-
Nitrate reduction	-
O/F test	Oxidation
Utilization of	
D-Glucose	+
D-Lactose	+
D-Maltose	+
D-Xylose	+
Sucrose	+
Starch	+

**Table 2:** The effects of different metal ions on enzyme activity.

Metal ion	Relative activity (%)	
	1 mM	10 mM
Control	100±2.5	100±2.5
K^+^	105.8±5.7	121.3±4.0
Ca^2+^	121.3±2.9	79.1±3.9
Mg^2+^	115.1±3.6	171.2±2.5
Al^3+^	88.8±3.7	76.9±3.7
Fe^2+^	82.5±2.7	68.3±2.4
Fe^3+^	98.4±2.1	87.9±4.4
Mn^2+^	56.2±2.2	33.7±4.7
Zn^2+^	91.2±2.1	101.4±4.4
Cu^2+^	46.6±3.4	24.1±2.1
Hg^2+^	44.9±6.7	36.9±4.3
Pb^2+^	112.3±2.0	73.1±4.1
Ba^2+^	119.4±3.0	78.9±6.5
Ni^2+^	139.4±2.9	150.2±3.1
Cd^2+^	71.3±2.8	56.4±4.9

**Table 3:** The effects of chemical reagents on enzyme activity.

Chemical reagents	Concentration	Relative activity (%)
Control	None	100±4.5
Tween 80	1%	50.8±3.8
	10%	48.4±6.3
Triton X-100	1%	79.1±4.2
	10%	69.9±4.1
DMSO	1%	95.7±4.3
	10%	141.9±5.4
SDS	1 mM	118.8±4.6
	10 mM	77.2±3.8
EDTA	1 mM	102.0±5.1
	10 mM	88.9±4.0
DTT	1 mM	84.9±5.1
	10 mM	74.7±4.0
*β*-ME	1 mM	75.8±5.1
	10 mM	31.9±4.7
Urea	1 mM	98.9±5.7
	10 mM	86.9±4.0

**Table 4:** Comparison of Amy175 with other well-characterized cold-active *α*-amylases.

Source and *α*-amylase	Molecular weight (kDa)/size	Identity with Amy175	Temperature optimum (°C)	Residual activity at 0°C	pH optimum	NaCl (M) for optimum activity	Activators	Inhibitors	Reference
*Pseudoalteromonas *sp. M175 (Amy175)	62/550 aa	-	25	53.2%	8.0	1	DMSO, Mg^2+^, Ni^2+^, K^+^ and 1 mM Ca^2+^, Ba^2+^, Pb^2+^	Cu^2+^, Hg^2+^, Mn^2+^, Tween 80, *β*-ME	This study
*Pseudoalteromonas haloplanktis *TAB23 (AHA)	49/453 aa	26%	25	20%	7.0	0.5	-	-	[21]
*Pseudoalteromonas arctica* GS230 (ParAmy)	55/477 aa	26%	30	34.5%	7.5	-	Mn^2+^, K^+^, Na^+^	Hg^2+^, Cu^2+^, Fe^3+^	[22]
*Pseudoalteromonas *sp. MY-1 (rAmyA)	73/669 aa	-	40	-	7.0	-	K^+^, Na^+^, Ca^2+^	-	[26]
*Zunongwangia Profunda *(AmyZ)	66/594 aa	26%	35	39%	7.0	1.5	Sr^2+^, Fe^3+^, Mg^2+^, Ba^2+^, NH_4_^+^, K^+^	Cu^2+^, Zn^2+^, Mn^2+^, Fe^3+^, SDS, EDTA	[23]
*Exiguobacterium *sp. SH3 (AmyE)	53/509 aa	24%	30	4%	6.5	2	Triton X-100, Tween 20	Acetone, DMSO, Butanol	[25]
uncultured organism (Amy_13C6_)	56/486 aa	22%	10-15	70% at 1°C	8.0-9.0	-	Ca^2+^	Cu^2+^, Zn^2+^, Ba^2+^, Mg^2+^, SDS, EDTA, Tween 20, Triton X-100	[27]
*Geomyces pannorum*	54/497 aa	-	40	over 20%	5.0	-	-	-	[40]
*Arthrobacter agilis *PAMC 27388	80/720 aa	-	30	-	3.0	-	Fe^3+^, *β*-ME	Co^2^^+^, ammonium persulphate (APS), SDS, Triton X-100, urea	[38]

**Table 5:** Comparison of Amy175 with other cold-active or thermophilic *α*-amylases.

*α*-amylase	Special feature	Optimal temperature (°C)	Arg (%)	Arg/(Arg+lys)ratio	Reference
Amy175	Cold active	25	2.7	0.32	This study
AHA	Cold active	25	2.9	0.5	[21]
ParAmy	Cold active	30	2.9	0.5	[22]
AmyZ	Cold active	35	3.5	0.36	[23]
Amy13	Mesophilic	60	4.6	0.62	[43]
Gt-amy	Thermophilic	80	4.0	0.4	[44]
AmyC	Thermophilic	90	5.4	0.42	[45]

The compositions of arginine and lysine in *α*-amylases were obtained from Genbank.

**Table 6:** Comparison of Amy175 with other halotolerant or halophile *α*-amylases.

*α*-amylase	Special feature	NaCl (M) for optimum activity	Asp+Glu (%)	Arg+lys (%)	Excess acidic amino acids (%)	Reference
Amy175	Halotolerant	1	13.3	8.3	5.0	This study
AHA	Halotolerant	0.5	10.4	5.7	4.7	[21]
AmyZ	Halotolerant	1.5	15.4	9.7	5.7	[23]
Amy13A	Halotolerant	0.86	16.7	11.9	4.8	[48]
AmyH	Halophile	4	16.5	6.1	10.4	[51]
*α*-amylase^A^	Haloalkaliphilic	2.5	24.3	5.4	18.9	[52]

The amino acid compositions of *α*-amylases were obtained from Genbank.

## Data Availability

The data used to support the findings of this study are available from the corresponding author upon request.
